# Data on the environmental impacts of the Hellisheiði geothermal plant and on the carbon intensity of geothermal energy and other energy technologies

**DOI:** 10.1016/j.dib.2019.104771

**Published:** 2019-11-07

**Authors:** Andrea Paulillo, Alberto Striolo, Paola Lettieri

**Affiliations:** Department of Chemical Engineering, University College London, Torrington Place, London, WC1 E7JE, United Kingdom

**Keywords:** Life cycle assessment, Carbon intensity, Environmental impacts, Geothermal energy

## Abstract

This data article is related to the research article “The environmental impacts and the carbon intensity of geothermal energy: A case study on the Hellisheiði plant”. The article reports numerical values of the results of the Life Cycle Assessment (LCA) study, which are reported only graphically and in an aggregated form in the main article. Data include normalised impacts, unaggregated environmental impacts of each life-cycle phase and activity in the foreground system, and results of Monte Carlo simulations. The article also includes data on the carbon intensity of other geothermal studies and alternative energy technologies, which were used for comparison in the associated research article.

**Table:** Specifications Table

Subject	Environmental Engineering
Specific subject area	Life Cycle Assessment and geothermal energy
Type of data	Table
How data were acquired	Data extracted from Gabi software and obtained from literature.
Data format	Raw (i.e. unaggregated) and processed (aggregated).
Parameters for data collection	Data for the Hellisheiði geothermal plant was generated by Gabi software; carbon intensities obtained from reliable literature sources.
Description of data collection	Material flows and life cycle inventory data for the Hellisheiði geothermal plant were collated from literature and LCA databases. Carbon intensities of geothermal energy and other energy technologies were obtained from literature.
Data source location	Iceland for the Hellisheiði geothermal plant, and worldwide for carbon intensities of geothermal energy and other energy technologies.
Data accessibility	With the article
Related research article	Paulillo, A. Striolo, P. Lettieri, The environmental impacts and the carbon intensity of geothermal energy: A case study on the Hellisheiði plant, Environ. Int. (2019)

**Table:** 

**Value of the Data** • Numerical values of the environmental impacts of the geothermal Hellisheiði plant and of the carbon intensity of geothermal energy and other energy technologies can be used for comparative purposes by other life-cycle studies. • Data is primarily of use to Life Cycle Assessment practitioners • Data provide the complete and unaggregated environmental impacts of each life-cycle phase and activity of the Hellisheiði plant.

## 1 Data

This article reports the complete, raw (unaggregated) and aggregated, life-cycle environmental impacts associated with the Hellisheiði geothermal plant in a double flash configuration, which are reported only in part and in graphical form in Paulillo et al. [1]. The study is based on the comprehensive life-cycle inventory developed by Karlsdóttir et al. [2]. Table 1 report the normalised environmental impacts per person in Europe calculated according to the ILCD (International reference Life Cycle Data system) method [3]. Table 2, Table 3, Table 4a, Table 4b, Table 5a, Table 5b, Table 6, Table 7, Table 8a, Table 8b, Table 9, Table 10, Table 11, Table 12, Table 13, Table 14, Table 15 report the contributions of materials and activities in the background system to each life-cycle phase and activity in the foreground system (the product system is reported in Ref. [1]). The environmental impacts reported in Table 1, Table 2, Table 3, Table 4a, Table 4b, Table 5a, Table 5b, Table 6, Table 7, Table 8a, Table 8b, Table 9, Table 10, Table 11, Table 12, Table 13, Table 14, Table 15 refer to a functional unit of 303 MJ electric and 133 MJ thermal, which correspond to the output from 1 s of operation of the plant.

**Table 1:** Normalised impacts of the hellisheiði ellisheidi geothermal plant.

Category	Unit	Normalisation factors [3,15]	Normalised impacts
Acidification	mole of H+ eq.	4.73E+01	2.75E-05
Climate change	kg CO_2_	9.22E+03	1.84E-04
Ecotoxicity - freshwater	CTUe	8.74E+03	8.91E-04
Eutrophication - freshwater	kg P eq.	1.48E+00	8.63E-05
Eutrophication - marine	kg N eq.	1.69E+01	2.40E-05
Eutrophication - terrestrial	mole of N Eq.	1.76E+02	2.27E-05
Human toxicity - cancer effects	CTUh	3.69E-05	2.69E-03
Human toxicity - non-cancer effects	CTUh	5.33E-04	3.41E-04
Land use	kg C deficit eq.	N/A	–
Ozone depletion	kg CFC-11 eq.	2.16E-02	6.64E-07
Particulate matter	kg PM2.5 eq.	3.80E+00	6.65E-05
Photochemical ozone formation	kg NMVOC eq.	3.17E+01	3.86E-05
Resource depletion - water	m3 eq.	8.14E+01	1.42E-05
Resource depletion - others	kg Sb eq.	1.01E-01	8.36E-05
Ionising radiations	Bq U235 air-equiv.	2.16E+05	9.61E-06

**Table 2:** Environmental impacts of the four main life cycle phases of the Hellisheiði geothermal plant.

Category	Unit	Construction	Operation	Maintenance	End of life	Total
Acidification	mole of H+ eq.	1.09E-03	0.00E+00	1.90E-04	1.65E-05	**1.30E-03**
Climate change	kg CO_2_	1.40E-01	1.53E+00	2.25E-02	2.45E-03	**1.70E** + **00**
Ecotoxicity - freshwater	CTUe	3.57E+00	0.00E+00	6.56E-01	3.57E+00	**7.79E** + **00**
Eutrophication - freshwater	kg P eq.	1.05E-04	0.00E+00	2.27E-05	3.26E-07	**1.28E-04**
Eutrophication - marine	kg N eq.	3.29E-04	0.00E+00	6.85E-05	7.41E-06	**4.05E-04**
Eutrophication - terrestrial	mole of N Eq.	3.25E-03	0.00E+00	6.80E-04	6.68E-05	**4.00E-03**
Human toxicity - cancer effects	CTUh	8.16E-08	0.00E+00	1.74E-08	1.29E-10	**9.92E-08**
Human toxicity - non-cancer effects	CTUh	1.51E-07	0.00E+00	2.91E-08	2.27E-09	**1.82E-07**
Land use	kg C deficit eq.	2.34E-01	0.00E+00	4.38E-02	1.61E-02	**2.93E-01**
Ozone depletion,	kg CFC-11 eq.	1.18E-08	0.00E+00	2.21E-09	3.74E-10	**1.43E-08**
Particulate matter	kg PM2.5 eq.	2.12E-04	0.00E+00	3.70E-05	3.70E-06	**2.53E-04**
Photochemical ozone formation	kg NMVOC eq.	9.87E-04	2.23E-05	1.97E-04	1.85E-05	**1.22E-03**
Resource depletion - water	m3 eq.	1.04E-03	0.00E+00	1.03E-04	1.07E-05	**1.15E-03**
Resource depletion - others	kg Sb eq.	7.60E-06	0.00E+00	7.76E-07	6.34E-08	**8.44E-06**
Ionising radiations	Bq U235 air-equiv.	1.80E+00	0.00E+00	2.51E-01	2.36E-02	**2.08E** + **00**

**Table 3:** Environmental impacts of the construction phase.

Category	Unit	Wells	Cogeneration plant	Collection pipelines	Total
Acidification	mole of H+ eq.	6.19E-04	3.38E-04	1.37E-04	**1.09E-03**
Climate change	kg CO_2_	6.83E-02	4.99E-02	2.23E-02	**1.40E-01**
Ecotoxicity - freshwater	CTUe	1.64E+00	1.31E+00	6.17E-01	**3.57E** + **00**
Eutrophication - freshwater	kg P eq.	5.36E-05	3.61E-05	1.50E-05	**1.05E-04**
Eutrophication - marine	kg N eq.	2.46E-04	5.88E-05	2.42E-05	**3.29E-04**
Eutrophication - terrestrial	mole of N Eq.	2.41E-03	5.71E-04	2.70E-04	**3.25E-03**
Human toxicity - cancer effects	CTUh	4.43E-08	2.09E-08	1.64E-08	**8.16E-08**
Human toxicity - non-cancer effects	CTUh	7.25E-08	5.40E-08	2.42E-08	**1.51E-07**
Land use	kg C deficit eq.	1.35E-01	6.87E-02	2.98E-02	**2.34E-01**
Ozone depletion,	kg CFC-11 eq.	7.47E-09	3.10E-09	1.18E-09	**1.18E-08**
Particulate matter	kg PM2.5 eq.	1.17E-04	6.53E-05	3.01E-05	**2.12E-04**
Photochemical ozone formation	kg NMVOC eq.	6.81E-04	2.12E-04	9.43E-05	**9.87E-04**
Resource depletion - water	m3 eq.	5.48E-04	3.65E-04	1.25E-04	**1.04E-03**
Resource depletion - others	kg Sb eq.	2.13E-06	4.50E-06	9.68E-07	**7.60E-06**
Ionising radiations	Bq U235 air-equiv.	6.91E-01	7.49E-01	3.64E-01	**1.80E** + **00**

**Table 4a:** Environmental impacts of construction of the geothermal wells.

Category	Unit	Diesel	Steel	Cement	Water	Aluminium	Bentonite	Lignosulfunite	Drilling waste disposal	Waste water treatment
Acidification	mole of H+ eq.	4.06E-04	1.72E-04	1.17E-05	3.10E-06	1.03E-05	1.75E-06	9.16E-07	3.20E-06	7.62E-06
Climate change	kg CO_2_	2.84E-02	3.08E-02	4.96E-03	4.86E-04	1.53E-03	1.99E-04	9.02E-05	4.12E-04	9.05E-04
Ecotoxicity - freshwater	CTUe	1.65E-02	1.19E+00	7.03E-03	7.00E-03	1.72E-02	2.44E-03	1.71E-03	3.73E-01	1.70E-02
Eutrophication - freshwater	kg P eq.	6.03E-07	2.74E-05	4.30E-07	3.49E-07	5.14E-07	4.34E-08	4.82E-08	2.26E-05	1.51E-06
Eutrophication - marine	kg N eq.	1.79E-04	3.20E-05	3.07E-06	4.98E-07	1.42E-06	4.84E-07	1.86E-07	1.09E-06	2.76E-05
Eutrophication - terrestrial	mole of N Eq.	1.96E-03	3.45E-04	3.58E-05	5.98E-06	1.44E-05	5.95E-06	1.90E-06	1.20E-05	2.12E-05
Human toxicity - cancer effects	CTUh	1.65E-10	3.45E-08	6.12E-11	2.19E-10	3.21E-10	1.07E-11	9.13E-12	8.80E-09	2.26E-10
Human toxicity - non-cancer effects	CTUh	9.50E-10	4.71E-08	4.06E-10	3.07E-10	3.68E-10	5.56E-11	6.33E-11	1.92E-08	3.95E-09
Land use	kg C deficit eq.	7.01E-02	3.84E-02	2.45E-03	6.04E-04	8.89E-04	5.63E-03	6.11E-04	9.45E-03	5.70E-03
Ozone depletion,	kg CFC-11 eq.	5.20E-09	1.78E-09	1.55E-10	4.63E-11	4.35E-11	2.73E-11	1.00E-11	1.15E-10	4.65E-11
Particulate matter	kg PM2.5 eq.	6.34E-05	4.88E-05	8.79E-07	4.17E-07	1.61E-06	1.85E-07	1.41E-07	3.59E-07	6.38E-07
Photochemical ozone formation	kg NMVOC eq.	5.13E-04	1.42E-04	8.84E-06	1.51E-06	4.37E-06	1.47E-06	5.44E-07	3.47E-06	3.00E-06
Resource depletion - water	m3 eq.	2.00E-05	2.32E-04	1.22E-05	2.59E-04	1.01E-05	1.16E-06	1.21E-06	2.36E-06	7.60E-06
Resource depletion - others	kg Sb eq.	1.45E-07	1.68E-06	3.59E-08	5.03E-08	2.30E-08	1.97E-08	8.69E-08	4.27E-08	2.95E-08
Ionising radiations	Bq U235 air-equiv.	1.03E-01	4.66E-01	2.39E-02	2.30E-02	4.67E-02	2.48E-03	2.38E-03	4.79E-03	1.41E-02

**Table 4b:** Environmental Impacts Of Construction Of The Geothermal Wells Cont'd

Category	Unit	Others^a^					Total
		Excavation	Perlite	Silica sand	Stainless steel	Concrete	
Acidification	mole of H+ eq.	1.13E-06	7.12E-08	7.17E-07	3.40E-08	1.03E-06	**6.19E-04**
Climate change	kg CO_2_	1.09E-04	4.75E-06	8.95E-05	5.11E-06	2.80E-04	**6.83E-02**
Ecotoxicity - freshwater	CTUe	2.13E-04	2.36E-05	4.21E-04	1.25E-04	9.70E-04	**1.64E** + **00**
Eutrophication - freshwater	kg P eq.	7.49E-09	1.06E-09	2.11E-08	1.98E-09	3.60E-08	**5.36E-05**
Eutrophication - marine	kg N eq.	4.83E-07	1.81E-08	1.32E-07	5.56E-09	2.75E-07	**2.46E-04**
Eutrophication - terrestrial	mole of N Eq.	5.29E-06	2.12E-07	1.44E-06	5.98E-08	3.07E-06	**2.41E-03**
Human toxicity - cancer effects	CTUh	5.26E-12	3.68E-13	3.94E-12	3.99E-12	7.69E-12	**4.43E-08**
Human toxicity - non-cancer effects	CTUh	8.62E-12	9.01E-13	1.78E-11	3.70E-12	4.29E-11	**7.25E-08**
Land use	kg C deficit eq.	2.59E-04	-3.80E-05	3.88E-04	8.60E-06	6.26E-04	**1.35E-01**
Ozone depletion,	kg CFC-11 eq.	1.92E-11	6.10E-13	6.91E-12	2.45E-13	1.64E-11	**7.47E-09**
Particulate matter	kg PM2.5 eq.	1.43E-07	6.21E-09	6.85E-08	1.39E-08	1.17E-07	**1.17E-04**
Photochemical ozone formation	kg NMVOC eq.	1.46E-06	5.39E-08	4.14E-07	1.89E-08	8.34E-07	**6.81E-04**
Resource depletion - water	m3 eq.	1.56E-07	2.23E-08	6.60E-07	2.89E-08	1.31E-06	**5.48E-04**
Resource depletion - others	kg Sb eq.	8.28E-10	3.89E-09	3.20E-09	7.12E-10	7.86E-09	**2.13E-06**
Ionising radiations	Bq U235 air-equiv.	5.43E-04	8.14E-05	1.98E-03	1.23E-04	2.02E-03	**6.91E-01**

a Others refer to the charts in the associated research article [1] where these activities were aggregated.

**Table 5a:** Environmental impacts of construction of the cogeneration plant.

Category	Unit	Copper	Asphalt	Reinforcing steel	Aluminium	Stainless steel	Concrete	Steel	Glass fibre reinforced plastic	Lubricating oil	Titanium
Acidification	mole of H+ eq.	7.45E-05	1.15E-05	8.39E-05	1.12E-05	3.35E-05	2.83E-05	2.87E-05	2.77E-05	2.33E-06	2.63E-05
Climate change	kg CO_2_	7.23E-04	1.38E-03	1.59E-02	1.71E-03	5.04E-03	7.67E-03	4.99E-03	6.16E-03	3.10E-04	4.65E-03
Ecotoxicity - freshwater	CTUe	5.88E-01	5.06E-03	2.79E-01	1.87E-02	1.23E-01	2.66E-02	2.12E-01	1.74E-02	2.23E-03	3.71E-02
Eutrophication - freshwater	kg P eq.	1.47E-05	3.80E-07	9.46E-06	5.82E-07	1.95E-06	9.88E-07	4.82E-06	5.75E-07	9.81E-08	2.33E-06
Eutrophication - marine	kg N eq.	5.97E-06	1.49E-06	1.55E-05	1.57E-06	5.49E-06	7.54E-06	5.39E-06	8.48E-06	3.16E-07	4.39E-06
Eutrophication - terrestrial	mole of N Eq.	4.50E-05	1.56E-05	1.67E-04	1.59E-05	5.89E-05	8.42E-05	5.80E-05	4.64E-05	3.37E-06	4.55E-05
Human toxicity - cancer effects	CTUh	9.36E-10	5.18E-11	8.88E-09	3.38E-10	3.94E-09	2.11E-10	5.96E-09	2.32E-10	1.61E-11	2.44E-10
Human toxicity - non-cancer effects	CTUh	2.79E-08	2.24E-10	1.04E-08	4.03E-10	3.65E-09	1.18E-09	8.64E-09	3.04E-10	9.27E-11	1.02E-09
Land use	kg C deficit eq.	2.78E-03	5.00E-03	1.77E-02	1.00E-03	8.47E-03	1.72E-02	6.49E-03	1.82E-03	1.94E-03	4.46E-03
Ozone depletion,	kg CFC-11 eq.	4.13E-11	2.76E-10	9.33E-10	5.16E-11	2.41E-10	4.51E-10	2.78E-10	1.13E-10	1.41E-10	4.71E-10
Particulate matter	kg PM2.5 eq.	6.40E-06	1.14E-06	2.07E-05	1.81E-06	1.37E-05	3.22E-06	8.34E-06	2.57E-06	2.91E-07	5.97E-06
Photochemical ozone formation	kg NMVOC eq.	1.27E-05	5.82E-06	7.74E-05	4.92E-06	1.86E-05	2.29E-05	2.31E-05	1.55E-05	5.88E-06	1.53E-05
Resource depletion - water	m3 eq.	8.86E-06	6.01E-06	1.02E-04	1.20E-05	2.85E-05	3.58E-05	2.79E-05	7.51E-05	2.66E-06	5.55E-05
Resource depletion - others	kg Sb eq.	1.34E-06	1.61E-08	4.69E-07	2.45E-08	7.03E-07	2.15E-07	3.06E-07	4.75E-08	5.38E-08	1.31E-06
Ionising radiations	Bq U235 air-equiv.	2.04E-02	3.93E-02	1.71E-01	5.23E-02	1.22E-01	5.55E-02	7.21E-02	3.55E-02	7.97E-03	1.66E-01

**Table 5b:** Environmental Impacts Of Construction Of The Cogeneration Plant Cont'd

Category	Unit	Others^a^				
		Excavation	Mineral wool	Polyethylene	PVC	Total
Acidification	mole of H+ eq.	4.41E-06	3.87E-06	1.21E-06	7.28E-07	**3.38E-04**
Climate change	kg CO_2_	4.26E-04	4.17E-04	2.90E-04	2.01E-04	**4.99E-02**
Ecotoxicity - freshwater	CTUe	8.32E-04	2.55E-03	5.86E-04	8.47E-04	**1.31E** + **00**
Eutrophication - freshwater	kg P eq.	2.93E-08	1.46E-07	5.48E-09	7.50E-09	**3.61E-05**
Eutrophication - marine	kg N eq.	1.89E-06	3.80E-07	2.10E-07	1.92E-07	**5.88E-05**
Eutrophication - terrestrial	mole of N Eq.	2.07E-05	6.32E-06	2.29E-06	1.98E-06	**5.71E-04**
Human toxicity - cancer effects	CTUh	2.05E-11	2.05E-11	9.50E-12	1.27E-11	**2.09E-08**
Human toxicity - non-cancer effects	CTUh	3.37E-11	9.78E-11	7.22E-12	2.92E-11	**5.40E-08**
Land use	kg C deficit eq.	1.01E-03	7.26E-04	4.45E-05	5.68E-05	**6.87E-02**
Ozone depletion,	kg CFC-11 eq.	7.48E-11	2.48E-11	2.08E-12	2.59E-12	**3.10E-09**
Particulate matter	kg PM2.5 eq.	5.58E-07	4.80E-07	7.20E-08	4.20E-08	**6.53E-05**
Photochemical ozone formation	kg NMVOC eq.	5.70E-06	1.87E-06	1.33E-06	1.00E-06	**2.12E-04**
Resource depletion - water	m3 eq.	6.09E-07	2.30E-06	7.88E-07	7.36E-06	**3.65E-04**
Resource depletion - others	kg Sb eq.	3.24E-09	7.77E-09	1.03E-09	1.37E-09	**4.50E-06**
Ionising radiations	Bq U235 air-equiv.	2.12E-03	4.62E-03	1.50E-04	1.68E-04	**7.49E-01**

a Others refer to the charts in the associated research article [1] where these activities were aggregated.

**Table 6:** Environmental impacts of construction of the collection pipelines.

Category	Unit	Aluminium	Excavation	Steel	Mineral wool	Concrete	Total
Acidification	mole of H+ eq.	2.95E-05	3.81E-06	7.36E-05	2.01E-05	9.65E-06	**1.37E-04**
Climate change	kg CO_2_	4.39E-03	3.68E-04	1.28E-02	2.17E-03	2.62E-03	**2.23E-02**
Ecotoxicity - freshwater	CTUe	4.92E-02	7.19E-04	5.45E-01	1.32E-02	9.10E-03	**6.17E-01**
Eutrophication - freshwater	kg P eq.	1.47E-06	2.53E-08	1.24E-05	7.61E-07	3.38E-07	**1.50E-05**
Eutrophication - marine	kg N eq.	4.06E-06	1.63E-06	1.39E-05	1.98E-06	2.58E-06	**2.42E-05**
Eutrophication - terrestrial	mole of N Eq.	4.12E-05	1.79E-05	1.49E-04	3.28E-05	2.88E-05	**2.70E-04**
Human toxicity - cancer effects	CTUh	9.18E-10	1.77E-11	1.53E-08	1.06E-10	7.21E-11	**1.64E-08**
Human toxicity - non-cancer effects	CTUh	1.05E-09	2.91E-11	2.22E-08	5.08E-10	4.02E-10	**2.42E-08**
Land use	kg C deficit eq.	2.54E-03	8.75E-04	1.67E-02	3.78E-03	5.87E-03	**2.98E-02**
Ozone depletion,	kg CFC-11 eq.	1.24E-10	6.46E-11	7.13E-10	1.29E-10	1.54E-10	**1.18E-09**
Particulate matter	kg PM2.5 eq.	4.61E-06	4.82E-07	2.14E-05	2.49E-06	1.10E-06	**3.01E-05**
Photochemical ozone formation	kg NMVOC eq.	1.25E-05	4.92E-06	5.94E-05	9.68E-06	7.81E-06	**9.43E-05**
Resource depletion - water	m3 eq.	2.89E-05	5.26E-07	7.15E-05	1.19E-05	1.22E-05	**1.25E-04**
Resource depletion - others	kg Sb eq.	6.58E-08	2.80E-09	7.85E-07	4.04E-08	7.37E-08	**9.68E-07**
Ionising radiations	Bq U235 air-equiv.	1.34E-01	1.83E-03	1.85E-01	2.40E-02	1.90E-02	**3.64E-01**

**Table 7:** Environmental impacts of the maintenance phase.

Category	Unit	Make-up wells	Additional collection pipelines	Total
Acidification	mole of H+ eq.	1.53E-04	3.42E-05	**1.87E-04**
Climate change	kg CO_2_	1.66E-02	5.59E-03	**2.22E-02**
Ecotoxicity - freshwater	CTUe	4.04E-01	1.54E-01	**5.58E-01**
Eutrophication - freshwater	kg P eq.	1.29E-05	3.75E-06	**1.67E-05**
Eutrophication - marine	kg N eq.	5.53E-05	6.02E-06	**6.13E-05**
Eutrophication - terrestrial	mole of N Eq.	6.05E-04	6.74E-05	**6.72E-04**
Human toxicity - cancer effects	CTUh	1.11E-08	4.10E-09	**1.52E-08**
Human toxicity - non-cancer effects	CTUh	1.72E-08	6.05E-09	**2.33E-08**
Land use	kg C deficit eq.	3.26E-02	7.44E-03	**4.00E-02**
Ozone depletion,	kg CFC-11 eq.	1.87E-09	2.96E-10	**2.17E-09**
Particulate matter	kg PM2.5 eq.	2.92E-05	7.53E-06	**3.67E-05**
Photochemical ozone formation	kg NMVOC eq.	1.72E-04	2.35E-05	**1.95E-04**
Resource depletion - water	m3 eq.	6.93E-05	3.13E-05	**1.01E-04**
Resource depletion - others	kg Sb eq.	5.17E-07	2.42E-07	**7.58E-07**
Ionising radiations	Bq U235 air-equiv.	1.55E-01	9.09E-02	**2.46E-01**

**Table 8a:** Environmental impacts of construction of the make-up wells.

Category	Unit	Diesel	Steel	Cement	Water	Aluminium	Bentonite	Lignosulfunite	Drilling waste disposal	Waste water treatment
Acidification	mole of H+ eq.	1.04E-04	4.37E-05	2.91E-06	7.44E-08	4.36E-07	2.29E-07	8.00E-07	8.00E-07	1.90E-06
Climate change	kg CO_2_	7.24E-03	7.84E-03	1.24E-03	8.86E-06	4.98E-05	2.25E-05	1.03E-04	1.03E-04	2.26E-04
Ecotoxicity - freshwater	CTUe	4.22E-03	3.03E-01	1.76E-03	3.03E-04	6.11E-04	4.27E-04	9.33E-02	9.33E-02	4.25E-03
Eutrophication - freshwater	kg P eq.	1.54E-07	6.99E-06	1.07E-07	1.65E-09	1.09E-08	1.21E-08	5.65E-06	5.65E-06	3.76E-07
Eutrophication - marine	kg N eq.	4.57E-05	8.17E-06	7.66E-07	2.73E-08	1.21E-07	4.64E-08	2.73E-07	2.73E-07	6.89E-06
Eutrophication - terrestrial	mole of N Eq.	5.00E-04	8.79E-05	8.94E-06	3.01E-07	1.49E-06	4.75E-07	3.00E-06	3.00E-06	5.29E-06
Human toxicity - cancer effects	CTUh	4.23E-11	8.81E-09	1.53E-11	6.01E-13	2.68E-12	2.28E-12	2.20E-09	2.20E-09	5.64E-11
Human toxicity - non-cancer effects	CTUh	2.43E-10	1.20E-08	1.01E-10	1.33E-11	1.39E-11	1.58E-11	4.81E-09	4.81E-09	9.87E-10
Land use	kg C deficit eq.	1.79E-02	9.79E-03	6.12E-04	7.37E-05	1.41E-03	1.53E-04	2.36E-03	2.36E-03	1.42E-03
Ozone depletion,	kg CFC-11 eq.	1.33E-09	4.55E-10	3.86E-11	1.74E-12	6.81E-12	2.51E-12	2.88E-11	2.88E-11	1.16E-11
Particulate matter	kg PM2.5 eq.	1.62E-05	1.25E-05	2.20E-07	2.07E-08	4.63E-08	3.53E-08	8.97E-08	8.97E-08	1.59E-07
Photochemical ozone formation	kg NMVOC eq.	1.31E-04	3.62E-05	2.21E-06	8.30E-08	3.66E-07	1.36E-07	8.66E-07	8.66E-07	7.50E-07
Resource depletion - water	m3 eq.	5.10E-06	5.94E-05	3.04E-06	4.53E-08	2.90E-07	3.01E-07	5.91E-07	5.91E-07	1.90E-06
Resource depletion - others	kg Sb eq.	3.70E-08	4.29E-07	8.97E-09	3.04E-10	4.91E-09	2.17E-08	1.07E-08	1.07E-08	7.38E-09
Ionising radiations	Bq U235 air-equiv.	2.64E-02	1.19E-01	5.96E-03	1.19E-04	6.20E-04	5.96E-04	1.20E-03	1.20E-03	3.52E-03

**Table 8b:** Environmental Impacts Of Construction Of The Make-Up Wells Cont'd.

Category	Unit	Excavation	Perlite	Silica sand	Stainless steel	Concrete	Total
Acidification	mole of H+ eq.	2.82E-07	1.78E-08	1.79E-07	8.48E-09	2.57E-07	**1.56E-04**
Climate change	kg CO_2_	2.73E-05	1.19E-06	2.24E-05	1.28E-06	6.99E-05	**1.70E-02**
Ecotoxicity - freshwater	CTUe	5.32E-05	5.90E-06	1.05E-04	3.13E-05	2.42E-04	**5.02E-01**
Eutrophication - freshwater	kg P eq.	1.87E-09	2.66E-10	5.28E-09	4.95E-10	9.00E-09	**1.90E-05**
Eutrophication - marine	kg N eq.	1.21E-07	4.53E-09	3.30E-08	1.39E-09	6.87E-08	**6.25E-05**
Eutrophication - terrestrial	mole of N Eq.	1.32E-06	5.30E-08	3.59E-07	1.49E-08	7.67E-07	**6.13E-04**
Human toxicity - cancer effects	CTUh	1.31E-12	9.20E-14	9.83E-13	9.97E-13	1.92E-12	**1.33E-08**
Human toxicity - non-cancer effects	CTUh	2.15E-12	2.25E-13	4.44E-12	9.25E-13	1.07E-11	**2.30E-08**
Land use	kg C deficit eq.	6.48E-05	-9.50E-06	9.70E-05	2.15E-06	1.56E-04	**3.64E-02**
Ozone depletion,	kg CFC-11 eq.	4.78E-12	1.52E-13	1.73E-12	6.11E-14	4.11E-12	**1.92E-09**
Particulate matter	kg PM2.5 eq.	3.57E-08	1.55E-09	1.71E-08	3.47E-09	2.94E-08	**2.95E-05**
Photochemical ozone formation	kg NMVOC eq.	3.64E-07	1.35E-08	1.04E-07	4.72E-09	2.08E-07	**1.73E-04**
Resource depletion - water	m3 eq.	3.89E-08	5.57E-09	1.65E-07	7.23E-09	3.26E-07	**7.18E-05**
Resource depletion - others	kg Sb eq.	2.07E-10	9.72E-10	7.99E-10	1.78E-10	1.96E-09	**5.35E-07**
Ionising radiations	Bq U235 air-equiv.	1.36E-04	2.03E-05	4.94E-04	3.08E-05	5.06E-04	**1.60E-01**

**Table 9:** Environmental impacts of construction of the additional collection pipelines for the make-up wells.

Category	Unit	Aluminium	Excavation	Steel	Mineral wool	Concrete	Total
Acidification	mole of H+ eq.	7.38E-06	9.51E-07	1.84E-05	5.02E-06	2.41E-06	**3.42E-05**
Climate change	kg CO_2_	1.10E-03	9.21E-05	3.20E-03	5.42E-04	6.55E-04	**5.59E-03**
Ecotoxicity - freshwater	CTUe	1.23E-02	1.80E-04	1.36E-01	3.31E-03	2.27E-03	**1.54E-01**
Eutrophication - freshwater	kg P eq.	3.68E-07	6.32E-09	3.10E-06	1.90E-07	8.44E-08	**3.75E-06**
Eutrophication - marine	kg N eq.	1.01E-06	4.07E-07	3.46E-06	4.94E-07	6.44E-07	**6.02E-06**
Eutrophication - terrestrial	mole of N Eq.	1.03E-05	4.46E-06	3.72E-05	8.21E-06	7.19E-06	**6.74E-05**
Human toxicity - cancer effects	CTUh	2.29E-10	4.43E-12	3.82E-09	2.66E-11	1.80E-11	**4.10E-09**
Human toxicity - non-cancer effects	CTUh	2.64E-10	7.27E-12	5.55E-09	1.27E-10	1.01E-10	**6.05E-09**
Land use	kg C deficit eq.	6.36E-04	2.19E-04	4.17E-03	9.44E-04	1.47E-03	**7.44E-03**
Ozone depletion,	kg CFC-11 eq.	3.11E-11	1.62E-11	1.78E-10	3.23E-11	3.85E-11	**2.96E-10**
Particulate matter	kg PM2.5 eq.	1.15E-06	1.20E-07	5.36E-06	6.22E-07	2.75E-07	**7.53E-06**
Photochemical ozone formation	kg NMVOC eq.	3.13E-06	1.23E-06	1.48E-05	2.42E-06	1.95E-06	**2.35E-05**
Resource depletion - water	m3 eq.	7.21E-06	1.31E-07	1.79E-05	2.98E-06	3.06E-06	**3.13E-05**
Resource depletion - others	kg Sb eq.	1.64E-08	6.99E-10	1.96E-07	1.01E-08	1.84E-08	**2.42E-07**
Ionising radiations	Bq U235 air-equiv.	3.34E-02	4.58E-04	4.63E-02	5.99E-03	4.74E-03	**9.09E-02**

**Table 10:** Environmental impacts of the end-of-life phase.

Category	Unit	Wells	CHP plant	Collection pipelines	Make-up wells	Additional collection pipelines	Total
Acidification	mole of H+ eq.	1.14E-06	1.08E-05	3.43E-06	2.89E-07	8.58E-07	**1.65E-05**
Climate change	kg CO_2_	2.86E-04	1.59E-03	4.07E-04	7.26E-05	1.02E-04	**2.45E-03**
Ecotoxicity - freshwater	CTUe	6.77E-03	3.50E+00	4.86E-02	1.69E-03	1.22E-02	**3.57E** + **00**
Eutrophication - freshwater	kg P eq.	3.53E-08	1.91E-07	7.28E-08	8.97E-09	1.82E-08	**3.26E-07**
Eutrophication - marine	kg N eq.	3.39E-07	5.44E-06	1.23E-06	8.57E-08	3.10E-07	**7.41E-06**
Eutrophication - terrestrial	mole of N Eq.	3.83E-06	4.50E-05	1.36E-05	9.71E-07	3.41E-06	**6.68E-05**
Human toxicity - cancer effects	CTUh	8.81E-12	7.80E-11	3.19E-11	2.23E-12	7.97E-12	**1.29E-10**
Human toxicity - non-cancer effects	CTUh	8.79E-11	1.49E-09	5.37E-10	2.21E-11	1.35E-10	**2.27E-09**
Land use	kg C deficit eq.	1.03E-03	9.09E-03	4.56E-03	2.59E-04	1.14E-03	**1.61E-02**
Ozone depletion,	kg CFC-11 eq.	2.07E-11	2.41E-10	8.63E-11	5.23E-12	2.15E-11	**3.74E-10**
Particulate matter	kg PM2.5 eq.	1.65E-07	2.43E-06	8.48E-07	4.15E-08	2.12E-07	**3.70E-06**
Photochemical ozone formation	kg NMVOC eq.	1.02E-06	1.24E-05	3.79E-06	2.58E-07	9.48E-07	**1.85E-05**
Resource depletion - water	m3 eq.	1.51E-06	6.15E-06	2.16E-06	3.83E-07	5.39E-07	**1.07E-05**
Resource depletion - others	kg Sb eq.	7.25E-09	3.61E-08	1.46E-08	1.84E-09	3.64E-09	**6.34E-08**
Ionising radiations	Bq U235 air-equiv.	2.22E-03	1.41E-02	5.37E-03	5.63E-04	1.34E-03	**2.36E-02**

**Table 11:** Environmental impacts of dismantling and closure of the geothermal wells.

Category	Unit	Aluminium disposal	Steel disposal	Concrete (not reinforced) disposal	Cement for closure	Gravel for closure	Total
Acidification	mole of H+ eq.	9.29E-09	7.01E-08	2.98E-07	4.89E-07	2.77E-07	**1.14E-06**
Climate change	kg CO_2_	1.41E-06	8.24E-06	3.55E-05	2.05E-04	3.59E-05	**2.86E-04**
Ecotoxicity - freshwater	CTUe	1.43E-04	4.85E-03	1.21E-03	3.06E-04	2.60E-04	**6.77E-03**
Eutrophication - freshwater	kg P eq.	2.47E-10	1.19E-09	6.61E-09	1.84E-08	8.89E-09	**3.53E-08**
Eutrophication - marine	kg N eq.	2.91E-09	2.35E-08	1.09E-07	1.28E-07	7.56E-08	**3.39E-07**
Eutrophication - terrestrial	mole of N Eq.	3.20E-08	2.58E-07	1.20E-06	1.49E-06	8.49E-07	**3.83E-06**
Human toxicity - cancer effects	CTUh	1.52E-13	1.13E-12	2.41E-12	2.62E-12	2.50E-12	**8.81E-12**
Human toxicity - non-cancer effects	CTUh	3.77E-12	3.48E-12	5.33E-11	1.73E-11	1.00E-11	**8.79E-11**
Land use	kg C deficit eq.	1.09E-05	1.92E-04	2.95E-04	1.05E-04	4.25E-04	**1.03E-03**
Ozone depletion,	kg CFC-11 eq.	2.69E-13	2.27E-12	6.97E-12	6.51E-12	4.69E-12	**2.07E-11**
Particulate matter	kg PM2.5 eq.	1.65E-09	7.76E-09	8.27E-08	3.75E-08	3.51E-08	**1.65E-07**
Photochemical ozone formation	kg NMVOC eq.	1.00E-08	7.39E-08	3.32E-07	3.69E-07	2.36E-07	**1.02E-06**
Resource depletion - water	m3 eq.	6.96E-09	4.91E-08	1.82E-07	5.15E-07	7.56E-07	**1.51E-06**
Resource depletion - others	kg Sb eq.	6.61E-11	3.57E-10	1.22E-09	1.58E-09	4.03E-09	**7.25E-09**
Ionising radiations	Bq U235 air-equiv.	1.36E-05	9.85E-05	4.78E-04	1.03E-03	6.08E-04	**2.22E-03**

**Table 12:** Environmental impacts of end-of-life of the cogeneration plant.

Category	Unit	Aluminium disposal	Copper disposal	Concrete (not reinforced) disposal		Mineral wool disposal	Asphalt disposal	Reinforcing steel disposal	Polyethylene disposal	PVC disposal	Inert waste disposal	Total
Acidification	mole of H+ eq.	2.49E-08	1.01E-08	7.14E-06	1.50E-08		5.43E-07	2.92E-06	3.98E-08	8.73E-08	8.88E-09	**1.08E-05**
Climate change	kg CO_2_	3.78E-06	1.54E-06	8.50E-04	1.51E-06		8.88E-05	2.86E-04	2.43E-04	1.10E-04	1.14E-06	**1.59E-03**
Ecotoxicity - freshwater	CTUe	3.85E-04	3.44E+00	2.91E-02	4.77E-06		2.85E-03	3.36E-03	1.56E-02	2.26E-03	2.63E-04	**3.50E** + **00**
Eutrophication - freshwater	kg P eq.	6.62E-10	2.70E-10	1.58E-07	1.84E-10		1.00E-08	1.65E-08	4.70E-10	4.22E-09	2.91E-10	**1.91E-07**
Eutrophication - marine	kg N eq.	7.80E-09	3.16E-09	2.61E-06	4.98E-09		1.40E-06	1.24E-06	1.03E-07	6.61E-08	2.95E-09	**5.44E-06**
Eutrophication - terrestrial	mole of N Eq.	8.57E-08	3.47E-08	2.88E-05	5.46E-08		1.89E-06	1.36E-05	1.89E-07	2.26E-07	3.23E-08	**4.50E-05**
Human toxicity - cancer effects	CTUh	4.09E-13	1.65E-13	5.77E-11	5.98E-14		4.43E-12	8.98E-12	1.63E-12	4.38E-12	2.51E-13	**7.80E-11**
Human toxicity - non-cancer effects	CTUh	1.01E-11	5.41E-11	1.28E-09	1.90E-13		1.92E-11	2.11E-11	7.07E-11	1.98E-11	1.21E-11	**1.49E-09**
Land use	kg C deficit eq.	2.91E-05	1.18E-05	7.08E-03	5.30E-05		8.08E-04	1.04E-03	1.50E-05	3.89E-05	1.22E-05	**9.09E-03**
Ozone depletion,	kg CFC-11 eq.	7.20E-13	2.93E-13	1.67E-10	5.12E-13		1.50E-11	5.27E-11	4.94E-13	3.46E-12	2.53E-13	**2.41E-10**
Particulate matter	kg PM2.5 eq.	4.43E-09	1.80E-09	1.98E-06	1.72E-09		6.62E-08	3.62E-07	2.09E-09	1.09E-08	1.05E-09	**2.43E-06**
Photochemical ozone formation	kg NMVOC eq.	2.69E-08	1.09E-08	7.96E-06	1.58E-08		5.55E-07	3.75E-06	4.88E-08	6.29E-08	9.22E-09	**1.24E-05**
Resource depletion - water	m3 eq.	1.87E-08	7.58E-09	4.35E-06	1.11E-08		4.09E-07	4.35E-07	2.47E-08	8.84E-07	6.80E-09	**6.15E-06**
Resource depletion - others	kg Sb eq.	1.77E-10	7.21E-11	2.91E-08	4.59E-11		2.27E-09	3.32E-09	9.99E-11	9.76E-10	4.84E-11	**3.61E-08**
Ionising radiations	Bq U235 air-equiv.	3.65E-05	1.48E-05	1.14E-02	1.81E-05		8.15E-04	1.53E-03	2.54E-05	2.61E-04	1.48E-05	**1.41E-02**

**Table 13:** Environmental impacts of end-of-life of the collection pipelines.

Category	Unit	Aluminium disposal	Steel disposal	Concrete disposal	Mineral wool disposal	Total
Acidification	mole of H+ eq.	2.66E-08	5.32E-07	2.79E-06	8.48E-08	**3.43E-06**
Climate change	kg CO_2_	4.03E-06	6.26E-05	3.32E-04	8.55E-06	**4.07E-04**
Ecotoxicity - freshwater	CTUe	4.10E-04	3.68E-02	1.14E-02	2.70E-05	**4.86E-02**
Eutrophication - freshwater	kg P eq.	7.05E-10	9.05E-09	6.20E-08	1.04E-09	**7.28E-08**
Eutrophication - marine	kg N eq.	8.31E-09	1.78E-07	1.02E-06	2.82E-08	**1.23E-06**
Eutrophication - terrestrial	mole of N Eq.	9.14E-08	1.95E-06	1.13E-05	3.08E-07	**1.36E-05**
Human toxicity - cancer effects	CTUh	4.36E-13	8.55E-12	2.26E-11	3.39E-13	**3.19E-11**
Human toxicity - non-cancer effects	CTUh	1.08E-11	2.64E-11	4.99E-10	1.07E-12	**5.37E-10**
Land use	kg C deficit eq.	3.10E-05	1.46E-03	2.77E-03	3.00E-04	**4.56E-03**
Ozone depletion,	kg CFC-11 eq.	7.68E-13	1.73E-11	6.53E-11	2.89E-12	**8.63E-11**
Particulate matter	kg PM2.5 eq.	4.71E-09	5.89E-08	7.75E-07	9.68E-09	**8.48E-07**
Photochemical ozone formation	kg NMVOC eq.	2.87E-08	5.61E-07	3.11E-06	8.96E-08	**3.79E-06**
Resource depletion - water	m3 eq.	1.99E-08	3.73E-07	1.70E-06	6.29E-08	**2.16E-06**
Resource depletion - others	kg Sb eq.	1.89E-10	2.71E-09	1.14E-08	2.60E-10	**1.46E-08**
Ionising radiations	Bq U235 air-equiv.	3.89E-05	7.48E-04	4.48E-03	1.02E-04	**5.37E-03**

**Table 14:** Environmental impacts of closure of the make-up wells.

Category	Unit	Aluminium disposal	Steel disposal	Concrete (not reinforced) disposal	Cement	Gravel	Total
Acidification	mole of H+ eq.	2.32E-09	1.75E-08	7.44E-08	1.24E-07	7.05E-08	**2.19E-06**
Climate change	kg CO_2_	3.52E-07	2.06E-06	8.86E-06	5.22E-05	9.14E-06	**2.99E-04**
Ecotoxicity - freshwater	CTUe	3.58E-05	1.21E-03	3.03E-04	7.80E-05	6.62E-05	**5.94E-03**
Eutrophication - freshwater	kg P eq.	6.16E-11	2.98E-10	1.65E-09	4.69E-09	2.27E-09	**3.85E-07**
Eutrophication - marine	kg N eq.	7.27E-10	5.86E-09	2.73E-08	3.26E-08	1.92E-08	**6.98E-06**
Eutrophication - terrestrial	mole of N Eq.	7.99E-09	6.43E-08	3.01E-07	3.81E-07	2.17E-07	**6.26E-06**
Human toxicity - cancer effects	CTUh	3.81E-14	2.81E-13	6.01E-13	6.67E-13	6.39E-13	**5.86E-11**
Human toxicity - non-cancer effects	CTUh	9.42E-13	8.69E-13	1.33E-11	4.41E-12	2.54E-12	**1.01E-09**
Land use	kg C deficit eq.	2.71E-06	4.80E-05	7.37E-05	2.67E-05	1.08E-04	**1.68E-03**
Ozone depletion,	kg CFC-11 eq.	6.72E-14	5.68E-13	1.74E-12	1.66E-12	1.20E-12	**1.68E-11**
Particulate matter	kg PM2.5 eq.	4.12E-10	1.94E-09	2.07E-08	9.55E-09	8.93E-09	**2.01E-07**
Photochemical ozone formation	kg NMVOC eq.	2.51E-09	1.85E-08	8.30E-08	9.40E-08	6.00E-08	**1.01E-06**
Resource depletion - water	m3 eq.	1.74E-09	1.23E-08	4.53E-08	1.31E-07	1.92E-07	**2.28E-06**
Resource depletion - others	kg Sb eq.	1.65E-11	8.91E-11	3.04E-10	4.01E-10	1.03E-09	**9.22E-09**
Ionising radiations	Bq U235 air-equiv.	3.40E-06	2.46E-05	1.19E-04	2.61E-04	1.55E-04	**4.08E-03**

**Table 15:** Environmental impacts of end-of-life of the additional collection pipelines for the make-up wells.

Category	Unit	Aluminium disposal	Steel disposal	Concrete disposal	Mineral wool disposal	Total
Acidification	mole of H+ eq.	6.64E-09	1.33E-07	6.97E-07	2.12E-08	**2.89E-07**
Climate change	kg CO_2_	1.01E-06	1.56E-05	8.31E-05	2.14E-06	**7.26E-05**
Ecotoxicity - freshwater	CTUe	1.03E-04	9.21E-03	2.85E-03	6.75E-06	**1.69E-03**
Eutrophication - freshwater	kg P eq.	1.76E-10	2.26E-09	1.55E-08	2.60E-10	**8.97E-09**
Eutrophication - marine	kg N eq.	2.08E-09	4.45E-08	2.56E-07	7.04E-09	**8.57E-08**
Eutrophication - terrestrial	mole of N Eq.	2.28E-08	4.89E-07	2.82E-06	7.71E-08	**9.71E-07**
Human toxicity - cancer effects	CTUh	1.09E-13	2.14E-12	5.64E-12	8.47E-14	**2.23E-12**
Human toxicity - non-cancer effects	CTUh	2.69E-12	6.60E-12	1.25E-10	2.68E-13	**2.21E-11**
Land use	kg C deficit eq.	7.75E-06	3.64E-04	6.91E-04	7.50E-05	**2.59E-04**
Ozone depletion,	kg CFC-11 eq.	1.92E-13	4.31E-12	1.63E-11	7.23E-13	**5.23E-12**
Particulate matter	kg PM2.5 eq.	1.18E-09	1.47E-08	1.94E-07	2.42E-09	**4.15E-08**
Photochemical ozone formation	kg NMVOC eq.	7.17E-09	1.40E-07	7.78E-07	2.24E-08	**2.58E-07**
Resource depletion - water	m3 eq.	4.97E-09	9.32E-08	4.25E-07	1.57E-08	**3.83E-07**
Resource depletion - others	kg Sb eq.	4.72E-11	6.77E-10	2.85E-09	6.49E-11	**1.84E-09**
Ionising radiations	Bq U235 air-equiv.	9.73E-06	1.87E-04	1.12E-03	2.55E-05	**5.63E-04**

This article also reports carbon intensities of geothermal energy and other energy technologies. Table 16 report the carbon intensities of Hellisheiði according to different configurations (i.e. single and double flash, and electricity-only production and co-generation of electricity and heat). Table 17, Table 18 report the carbon intensities of geothermal plants estimated by other LCA studies [[4], [5], [6], [7], [8], [9], [10], [11], [12], [13]] and of alternative energy technologies as reported by the IPCC [14]. The carbon intensity is defined as the life-cycle emission of greenhouse gases expressed in terms of CO2 equivalents (i.e. in terms of their potential to contribute to global warming) per kWh of electricity produced.

**Table 16:** Results of Monte Carlo simulations for single (Sf) and double (df) flash Configurations, and for combined heat and power (chp), according to energy, exergy or price-based allocation, and power only Configurations.

			10%	25%	Median	75%	90%
**Single flash**	CHP	Energy	12.89	15.53	18.40	21.48	24.34
		Exergy	13.26	15.98	18.93	22.10	25.05
		Price	18.49	22.28	26.39	30.82	34.93
	Power only		18.84	22.51	26.82	31.13	35.44
**Double flash**	CHP	Energy	10.97	13.23	15.76	18.39	21.01
		Exergy	11.47	13.83	16.48	19.22	21.97
		Price	16.46	19.84	23.64	27.58	31.52
	Power only		16.63	19.90	23.73	27.71	31.27

**Table 17:** Carbon intensity of other geothermal energy plants.

Technology	Source	gCO_2_ eq.
Dry steam	Buonocore et al., 2015	248
Single Flash	Bravi and Basosi, 2014	776
Double flash	Hondo, 2005	15
Double flash	Atilgan and Azapagic, 2016	63
Double flash	Marchand et al., 2015	47
Binary	Martin Gamboa et al., 2015	2
Binary	Frick et al., 2010	52
Binary	Lacirignola and Blanc, 2013	37
Binary	Pratiwi et al., 2018	25
Binary	Rule et al., 2009	5.6

Notes:^1^ Scenario A1, ^2^ Base case (Scenario 6), ^3^ Scenario S2.

**Table 18:** Carbon Intensity Of Selected Energy Sources From Ipcc [14].

	Min	Median	Max
Coal (PC)	740	820	910
Gas (combined cycle)	410	490	650
Geothermal	6	38	79
Hydropower	1	24	2200
Nuclear	3.7	12	110
Solar (PV)	18	48	180
Wind (onshore)	7	11	56

## 2 Experimental design, materials, and methods

The environmental impacts of Hellisheiði were generated with Gabi sustainability software, version 8, using the life-cycle inventory developed by Karlsdóttir et al. [2] for the foreground system and the Ecoinvent database version 3.4 [16] for all background activities; the ILCD impact method [17] enhanced with the radiological impact category for ionising radiations developed by Paulillo [15] was used to translate the inventory into environmental impacts. Numerical values of impacts generated by the LCA software have been only slightly amended to improve readability and clarity.

Monte Carlo simulations were performed for single- and double-flash configurations and for the case of electricity-only production and co-generation of heat and power. The Monte Carlo simulations were performed in Gabi with a number of iterations equal to 10,000.

The data on carbon intensities of geothermal energy and other energy technologies were collated from literature and are reported unchanged here.
